# The clinical, electrocardiographic and echocardiographic characteristics and long-term outcome of patients with tachycardia-induced cardiomyopathy

**DOI:** 10.5830/CVJA-2011-019

**Published:** 2012-04

**Authors:** Ashley Chin, Motasim Badri, Nb Ntusi, Andrzej Okreglicki

**Affiliations:** Cardiac Clinic, Groote Schuur Hospital, and Department of Medicine, University of Cape Town, Cape Town, South Africa; Cardiac Clinic, Groote Schuur Hospital, and Department of Medicine, University of Cape Town, Cape Town, South Africa; Cardiac Clinic, Groote Schuur Hospital, and Department of Medicine, University of Cape Town, Cape Town, South Africa; Cardiac Clinic, Groote Schuur Hospital, and Department of Medicine, University of Cape Town, Cape Town, South Africa

**Keywords:** tachycardia-induced cardiomyopathy, cardiomyopathy, tachycardia, tachycardiomyopathy, tachycardia-mediated cardiomyopathy

## Abstract

**Introduction:**

The clinical, electrocardiographic and echocardiographic features and long-term outcome of patients with tachycardia-induced cardiomyopathy (TIC) have not been well described in the past.

**Methods:**

A retrospective study was performed at our institution of patients with a diagnosis of TIC.

**Results:**

Thirty-three patients with pure TIC and 12 patients with impure TIC were identified. Compared to patients with dilated cardiomyopathy (DCMO), pure TIC patients were less symptomatic, as judged by NYHA class (*p* = 0.02), they had fewer clinical signs of heart failure (*p* = 0.007) and were more likely to report palpitations (*p* = 0.007) at presentation. Electrocardiographically, pure TIC patients had fewer Q waves (*p* = 0.002), less left ventricular hypertrophy (LVH) (*p* = 0.004) and repolarisation abnormalities (*p* = 0.048), and shorter QRS durations (*p* = 0.024). Echocardiographically, pure TIC patients had significantly smaller left ventricular internal diameter in diastole (LVIDd) (*p* < 0.001), ventricular internal diameter in systole (LVIDs) (*p* = 0.001) and left atrial dimensions (*p* = 0.048) at presentation compared to DCMO patients. Patients with pure TIC had a trend towards increased residual LVIDd dimensions compared to a control group with normal echocardiograms, indicating a persistence of adverse LV remodelling late after control of the causative tachycardia (*p* = 0.06). Recurrent tachycardia occurred in three patients, which resulted in a precipitous decline in left ventricular ejection fraction (LVEF).

**Conclusions:**

This study is the first to compare features of pure and impure TIC. Patients with pure TIC had shorter QRS durations, fewer Q waves, and less LVH and repolarisation abnormalities at presentation compared to DCMO patients. TIC patients tended to have smaller LVIDd dimensions at presentation and have persistence of adverse LV remodelling, as characterised by persistent enlargement of LVIDd dimensions, at late follow up.

Tachycardia-induced cardiomyopathy (TIC) is an important reversible cause of cardiomyopathy (CMO) and heart failure. Until recently, TIC was considered to be a rare cause of reversible left ventricular (LV) dysfunction.^1^ Over the past few years, several publications have established that this disease is much more prevalent than once thought,^2^,^03^ but most reports of TIC have been isolated case reports or small retrospective cohorts comprising less than 20 to 30 patients.

LV dysfunction may either develop in the setting of no underlying structural heart disease, so-called ‘pure’ TIC, or in the setting of pre-existing structural heart disease, ‘impure’ TIC.^04^ TIC studies have mostly enrolled patients with pure TIC and have excluded those with impure TIC. However, impure TIC is probably more common than pure TIC,^04^ but less well investigated and reported in the literature.

The presenting clinical, electrocardiographic and echocardiographic features of TIC have not been well described. At presentation, TIC may be indistinguishable from dilated cardiomyopathy (DCMO) with secondary tachycardia – the so-called ‘chicken–egg dilemma’.^05^ Factors influencing the rate and extent of recovery of LV function are poorly understood. Lastly, the long-term outcome and prognosis of patients with TIC have not been well defined.

In order to examine some of these questions, we conducted a retrospective study of TIC at our institution. In this study, we report the clinical, electrocardiographic and echocardiographic features of a relatively large cohort of 45 patients with pure and impure TIC. One of the aims of this study was to identify clinical, electrocardiographic and echocardiographic features that could help the clinician recognise this condition, by comparing 25 patients with pure TIC with 25 patients with DCMO. We report on the response to treatment and compare 17 patients who had normalised LV function after control of the tachycardia with 17 control patients with normal echocardiograms, to assess for persistence of adverse LV remodelling. Finally, we report on the long-term outcome and prognosis of these patients.

## Methods

We conducted a retrospective study of patients with a diagnosis of TIC who presented to the cardiac clinic at Groote Schuur Hospital, Cape Town between 1994 and 2009. The study protocol was approved by the University of Cape Town’s ethics committee.

We included patients with a diagnosis of TIC made by the attending cardiologist if the presenting left ventricular ejection fraction (LVEF) was < 50% and there was an LVEF improvement of ≥ 5% after rate or rhythm control of the tachycardia with medical or ablative therapy. Patients were included in the pure TIC group if no underlying structural heart disease could be identified. Patients with a prior history of underlying structural heart disease were included in the impure TIC group and analysed separately. Patients with a diagnosis of hypertension were included in the impure TIC group.

The cohort of 33 patients with pure TIC was compared to 12 patients with impure TIC. We were able to compare 25 patients with pure TIC with 25 control patients with DCMO (matched for age, gender and presenting LVEF) with regard to clinical, electrocardiographic and echocardiographic parameters. The DCMO patients were obtained from the CMO registry at Groote Schuur Hospital (unpublished data).

To assess for persistence of adverse LV remodelling after recovery of LV function, the last available echocardiograms of 17 patients with pure TIC who had recovered LV function (post-treatment LVEF ≥ 50%) were compared to normal echocardiograms of 17 patients matched for age and LVEF. For the purposes of this article, TIC includes pure and impure TIC unless otherwise specified.

Retrospective data collection included clinical, electrocardiographic and echocardiographic characteristics and long-term follow up of patients diagnosed with pure or impure TIC and matched DCMO controls. Patients’ clinical records were extensively reviewed. All previous electrocardiograms (ECGs) of patients with TIC were retrieved from an ECG archival system (MUSE CV system) and reviewed to calculate mean heart rate prior to the diagnosis of TIC. All 24-hour Holter monitors performed prior to and at the time of the diagnosis of TIC were analysed to determine mean and maximum heart rates during a 24-hour period.

Transthoracic echocardiography was performed to measure LVEF, left ventricular internal diameter in diastole (LVIDd), left ventricular internal diameter in systole (LVIDs), left atrial (LA) size, and to quantitate the severity of mitral regurgitation (MR) using standard M-mode or modified Simpson’s method according to the recommendations of the American Society of Echocardiography. Radionucleotide imaging that calculated LVEF was used when available. Serial echocardiograms, if available, were reviewed in patients with TIC to assess the rate and magnitude of LV functional improvement. All subsequent visits were reviewed and recurrences of any tachycardias were documented. Patients who had been discharged to follow up or who were no longer attending the cardiac clinic were telephonically contacted.

The choice of treatment of the causative tachycardia was at the discretion of the attending cardiologist. Generally, in patients with atrial fibrillation (AF), rate control using atrio-ventricular (AV) nodal blockers (beta-blockers, calcium channel blockers), anti-arrhythmic drugs or Digoxin was initially preferred. Patients with drug-resistant AF who continued to have poor rate control despite the above treatment, were referred for AV node ablation (AVNA) and permanent pacemaker (PPM) implantation. Catheter ablation was considered first-line therapy for certain arrhythmias [atrial flutter (AFL), atrial tachycardia (AT), atrio-ventricular nodal re-entry tachycardia (AVNRT), atrio-ventricular re-entry tachycardia (AVRT) and fascicular ventricular tachycardia (VT)].

## Statistical analysis

Results are expressed as mean (standard deviation) or median (interquartile range). Clinical and socio-demographic characteristics of the groups studied were compared using the Student’s *t*-test, Chi-square test, Mann-Whitney test or Wilcoxon signed rank test, whichever was appropriate. A *p*-value of < 0.05 was considered significant. Non-parametric tests were used due to the non-normality of some of the variables studied. Receiver operating characteristic (ROC) curves were plotted to examine the diagnostic utility of some variables, which was expressed as an area under the curve and measured using the *C*-statistic. Data were analysed using SPSS version 17 (Chicago, Illinois; 2009).

## Results

Patient demographics, clinical features, investigations, treatment and long-term outcomes of the pure and impure TIC cohort are displayed in Table 1.

**Table 1:** Cohort Of Pure And Impure Tic Patients

*Patient*	*Age (years)*	*Gender (M/F)*	*NYHA (Class)*	*Dyspnoea duration (months*	*Paipitations (yes/no)*	*Palpitations duration (months)*	*Rhythm*	*Heart rate (bpm)*	**Initial LVEF	*Last LVEF*	*Treatment*	*Recurrent TIC*	*Alive*
Pure TIC													
Patient 1	30	F	3	1	Yes	1	AT	197	14%	53%	Medical Rx	Medical Rx	
Patient 2	20	M	2	2	Yes	2	AT	136	42%	47%	AT ablation	No	
Patient 3	62	M	3	1	Yes	1	AF	89	30%	50%	AVNA/PPM	No	No
Patient 4	30	F	3	2	Yes	8	AT	128	16%	53%	Medical Rx	Yes	Yes
Patient 5	68	M	3		Yes	6	AFL	148	35%	85%	AFL ablation	No	Yes
Patient 6	42	M	3	2	Yes	60	AFL	102	38%	60%	AFL ablation	No	Yes
Patient 7	52	F	2	3	Yes	3	AF	156	45%	54%	Medical Rx	No	Yes
Patient 8	46	M	3	96	No		AF	100	21%	36%	AVNA/PPM	No	Yes
Patient 9	45	M	1	0	Yes	240	AFL/AF	102	49%	67%	AVNA/PPM	No	Yes
Patient 10	40	M	2	3	Yes	3	AFL	165	13%	63%	AVNA/PPM/AFL ablation	No	Yes
Patient 11	54	M	3	12	Yes		AF, previous AFL	84	22%	*	Medical Rx,AFL ablation	No	Yes
Patient 12	28	M	2	1	Yes	1	AF	123	27%	35%	AVNA/PPM	No	
Patient 13	38	M	4		Yes		AF		30%	63%	Medical Rx	No	
Patient 14	68	F	3	1	Yes	1	AVNRT	132	30%	40%	AVNRT ablation	No	Yes
Patient 15	17	F	3	2	Yes	2	AT	131	23%	40%	Medical Rx	No	Yes
Patient 16	47	F	3	12	Yes	24	AF	126	48%	59%	AVNA/PPM	No	Yes
Patient 17	56	M	2	9	Yes	3	AFL	123	33%	64%	AFL ablation	No	Yes
Patient 18	33	F	1	0	No		AF/WPW	240	33%	41%	WPW ablation	No	
Patient 19	41	M	3	1	No		AF	133	22%	49%	AVNA/PPM	No	Yes
Patient 20	65	M	4	8	No	0	AF	136	23%	60%	AVNA/PPM	No	No
Patient 21	46	M	3	1	Yes	11	AFL	144	31%	51%	AFL ablation	No	Yes
Patient 22	79	F	3	1	No	0	AF	167	30%	50%	Medical Rx/AVNA/PPM	No	
Patient 23	38	M	1	0	Yes	6	AF/AFL	82	40%	53%	Medical Rx/AFL ablation	Yes	
Patient 24	16	M	4	0	Yes	0	Fascicular VT	178	29%	56%	VT ablation	No	Yes
Patient 25	47	M	3	12	Yes	108	AFL	95	37%	58%	AFL ablation/medical Rx	No	Yes
Patient 26	64	M	3	6	Yes	72	AFL/AF	105	37%	50%	AFL ablation/medical Rx	No	Yes
Patient 27	50	M	2		Yes	60	AF	100	41%	50%	AVNA/PPM	No	
Patient 28	71	M	3	24	No	0	AF/AFL	130	35%	41%	AFL ablation	No	
Patient 29	62	M	4	3	Yes	120	AF	133	39%	45%	AVNA/PPM	No	Yes
Patient 30	57	M	3	36	Yes	144	AF/AFL	102	40%	50%	AVNA/PPM	No	
Patient 31	48	M	1	0	Yes		AFL	150	42%	65%	AFL ablation	No	Yes
Patient 32	55	M	2		Yes	6	AF	86	35%	49%	Medical Rx	No	Yes
Patient 33	66	M	3	12	Yes	12	AF	93	38%	64%	AVNA/PPM	No	No
Impure TIC													
Patient 34	37	F	2		Yes	0	AFL	66	38%	43%	AVNA/PPM	No	Yes
Patient 35	53	M	3				AFL	96	35%	48%	Medical Rx	No	
Patient 36	62	M	3	1	No	6	AF/AFL	102	31%	47%	Medical Rx	No	
Patient 37	40	F	4	0	No		Atypical AVNRT	72	27%	64%	AVNRT ablation	No	Yes
Patient 38	52	M	4	0			AF		48%	67%	AVNA/PPM	No	Yes
Patient 39	63	M	3		Yes	144	AF	114	42%	54%	AVNA/PPM	No	Yes
Patient 40	35	M	3		Yes	12	AF	126	30%	47%	AVNA/PPM	No	Yes
Patient 41	56	M	3	0	Yes	3	AVRT	94	21%	48%	AVRT ablation	Yes	Yes
Patient 42	20	M	3	1	Yes	1	Atypical AFL	72	20%	51%	Medical Rx	No	Yes
Patient 43	39	M	4	3	Yes	3	AF/AFL	136	19%	64%	AFL ablation	No	
Patient 44	25	F	4	0	Yes	0	AFL	78	17%	72%	AVNA/PPM	No	Yes
Patient 45	28	M	4	1	Yes	1	AVNRT	84	22%	*	AVNRT ablation	No	

AF: atrial fibrillation, AFL: atrial flutter, AT: atrial tachycardia, AVNRT: atrio-ventricular nodal re-entrant tachycardia, AVRT: atrio-ventricular re-entrant tachycardia, AVNA: atrio-ventricular node ablation, LVEF: left ventricular ejection fraction, NYHA: New York Heart Association, PPM: permanent pacemaker, Rx: treatment, VT: ventricular tachycardia, WPW: Wolff-Parkinson-White syndrome; * LVEF not documented, but improvement noted; blank space = unknown.

The pure TIC cohort consisted of 33 patients (25 males, eight females). The median age was 44 (30–62) years with a marked male predominance (76%). Twenty-one (64%) patients presented with severe effort intolerance [New York Heart Association (NYHA) class III, IV]. The median duration of dyspnoea and palpitations prior to presentation was two (0.6–7.5) months and seven (1.3–51.0) months, respectively. The arrhythmic causes were AF (*n* = 20), AFL (*n* = 7), AT (*n* = 4), AVNRT (*n* = 1) and fascicular VT (*n* = 1). The most common ECG abnormalities were repolarisation abnormalities (55%), followed by LV hypertrophy (12%) and LA hypertrophy (9%). Q waves, left bundle branch block (LBBB) and right bundle branch block (RBBB) were seen in less than 7% of presenting ECGs. The mean LVEF at presentation was 32.4 ± 9.5%. The mean LVIDd and LVIDs dimensions were 5.7 ± 0.7 and 4.8 ± 0.8 cm, respectively. The mean LA size was 4.2 ± 1.0 cm.

The impure TIC cohort consisted of 12 patients (nine males, three females). Patients had a history of hypertension (*n* = 3), viral myocarditis (*n* = 2), valvular heart disease (*n* = 3), ischaemic heart disease (*n* = 2), patent ductus arteriosus (*n* = 1) and peripartum cardiomyopathy (*n* = 1). The median age was 39 (23–59) years with a marked male predominance (75%). Eleven (92%) patients presented with severe effort intolerance (NYHA III, IV). The median duration of dyspnoea and palpitations prior to presentation was 0.5 (0.2–2.0) months and 3 (0.6–4.5) months, respectively. The arrhythmic causes were AF (*n* = 5), AFL (*n* = 4), AVNRT (*n* = 2) and AVRT (*n* = 1). The most common ECG abnormalities were repolarisation abnormalities (70%) and LV hypertrophy (70%). The mean LVEF at presentation was 29.2 ± 1.0%. The mean LVIDd and LVIDs dimensions were 5.7 ± 1.0 cm and 4.8 ± 0.8 cm, respectively. The mean LA size was 4.8 ± 1.5 cm.

Compared to patients with pure TIC, patients with impure TIC had shorter durations of dyspnoea (*p* = 0.04) and more clinical signs of heart failure at presentation (*p* = 0.003). Patients with impure TIC displayed more ECG features of underlying structural heart disease [LA hypertrophy (*p* = 0.05), LV hypertrophy (*p* < 0.001) and larger RV^06^ voltages (*p* = 0.04)]. There were no significant differences in presenting echocardiographic features between the two groups.

In order to identify characteristics that may be useful to differentiate between pure TIC and DCMO at presentation, we compared 25 patients with pure TIC with 25 patients with DCMO, matched for age, gender and LVEF. The clinical, electrocardiographic and echocardiographic features of the two groups are displayed in Table 2.

**Table 2:** Comparison Of Clinical, Electrocardiographic And Echocardiographic Features Of Pure Tic And Dcmo Matched For Age, Gender And LVEF

*Patient demographics and clinical features*	*Pure TIC group (n = 25) n (%)*	*DCMO group (n = 25) n (%)*	p-value*
Age	46 (16–67)	46 (21–65)	0.846^¶^
Gender			
Male	19 (76)	19 (76)	
Female	6 (24)	6 (24)	1.00
NYHA			
NYHA I, II	9 (36)	4 (16)	
NYHA III, IV	16 (64)	21 (84)	0.02
Palpitations			
Yes	21 (84)	12 (48)	
No	4 (16)	13 (52)	0.007
^¶^Mann-Whitney test, *Chi-square test, ^#^Student’s *t*-test.			
Signs of heart failure			
Yes	12 (48)	21 (84)	
No	13 (52)	4 (16)	0.007
ECG features			
Sinus rhythm			
Yes	5 (20)	19 (76)	
No	20 (80)	6 (24)	< 0.001
LA enlargement			
Yes	3 (12)	8 (32)	
No	5 (20)	11 (44)	0.546
Unable to assess	17 (68)	6 (24)	
Q waves			
Yes	1 (4)	10 (40)	
No	24 (96)	15 (60)	0.002
LBBB			
Yes	2 (8)	6 (24)	
No	23 (92)	19 (76)	0.123
RBBB			
Yes	1 (4)	0 (0)	
No	24 (96)	25 (100)	0.5
LVH			
Yes	4 (16)	14 (56)	
No	21 (84)	11 (44)	0.004
Repolarisation abnormalities			
Yes	16 (64)	22 (88)	
No	9 (36)	3 (12)	0.048
QRS width (ms)	88 (82-102)	100 (92-110)	0.024^¶^
RV_6_ (mm)	15.8 ± 7.5	15.0 ± 11.2	0.79^#^
RV_6_/R_max_	1.84 ± 0.8	1.69 ± 1.2	0.63^#^
Echocardiographic features			
LVEF (%)	30.8 ± 9.5	27.8 ± 10.2	0.29^#^
LVIDd (cm)	5.7 ± 0.7	6.6 ± 0.6	< 0.001^#^
LVIDs (cm)	4.9 ± 0.8	5.7 ± 0.6	0.001^#^
LA size (cm)	4.1 ± 1.0	4.6 ± 0.5	0.048^#^

Clinically, pure TIC patients were less symptomatic according to NYHA class (*p* = 0.02), had fewer clinical signs of heart failure (*p* = 0.007) and were more likely to report palpitations (*p* = 0.007) at presentation. Electrocardiographically, TIC patients had fewer Q waves (*p* = 0.002), less LV hypertrophy (*p* = 0.004) and repolarisation abnormalities (*p* = 0.048), and shorter QRS durations (*p* = 0.024). Six DCMO patients had underlying paroxysmal or permanent AF. There were no significant differences in the prevalence of LA enlargement, LBBB, RBBB, RV_6_ voltage or RV_6_/RV_max_ ratio between the two groups. Echocardiographically, pure TIC patients had significantly smaller LVIDd (*p* < 0.001), LVIDs (*p* = 0.001) and LA dimensions (*p* = 0.048) compared to DCMO patients at presentation.

Despite significant differences in LVIDd dimensions and QRS duration between the two groups, we could not identify any LVIDd dimension [area under the receiver operating curve (ROC) = 0.1989] or QRS duration (ROC = 0.2558) that could predict a diagnosis of pure TIC with a high sensitivity and specificity.

The pre- and post-treatment echocardiographic parameters of pure and impure TIC are displayed in Table 3. In the pure TIC group, the mean LVEF improved significantly from 32.4 ± 9.5 to 53.2 ± 10.5% (*p* < 0.001). Both the LVIDd (*p* = 0.004) and LVIDs (*p* = 0.001) dimensions improved significantly post treatment. There was no significant decrease in LA size pre-and post treatment. In the impure TIC group, the mean LVEF improved significantly from 29.2 ± 10.0 to 55.0 ± 9.9% (*p* < 0.001). The LVIDs dimension (*p* = 0.04), but not the LVIDd dimension, improved significantly post treatment. There was no change in LA size pre- and post treatment in the impure TIC group. TIC patients with dilated ventricles at presentation were more likely to have residual LV dilatation at follow up (*r* = 0.68, *p* = 0.002).

**Table 3:** Pre- And Post-Treatment Echocardiographic Parameters Of Pure And Impure TIC

	*Pure TIC at presentation (n = 33)*	*Pure TIC post-treatment (n = 33)*	p-*value^#^*	*Impure TIC at presentation (n = 12)*	*Impure TIC post-treatment (n = 12)*	p-*value^#^*
LVEF (%)	32.4 ± 9.5	53.2 ± 10.5	< 0.001	29.2 ± 10.0	55.0 ± 9.9	< 0.001
LVIDd (cm)	5.7 ± 0.7	5.3 ± 0.8	0.004	5.7 ± 1.0	5.5 ± 0.8	0.86
LVIDs (cm)	4.8 ± 0.8	3.6 (2.2-4.6)	0.001^¶^	4.8 ± 0.8	3.8 (2.7-4.4)	0.04^¶^
LA size (cm)	4.2 ± 1.0	4.1 ± 0.8	0.19	4.81 ± 1.5	4.7 ± 1.3	0.92

^#^Student’s *t*-test, ^¶^Mann-Whitney test.

We identified variable rates of LV improvement after control of the tachycardia. Thirteen pure TIC patients had at least three echocardiograms performed: at initial diagnosis, when improvement of LV function was documented, and at last available follow up. In seven (54%) patients, maximal recovery was noted early, within the first three to six months after control of the tachycardia (Fig. 1: group A). All seven patients had prompt, effective rate or rhythm control of the causative tachycardia. However, in six (46%) patients, maximal LV improvement occurred late, after six months, with improvement seen even after a year (Fig. 2: group B). Of these six patients, two had LVEF < 20%. Two AF patients had initial suboptimal heart rate control as defined by the AFFIRM trial targets^06^ before AVNA and PPM implantation, and two patients had ATs that were initially difficult to control with medical therapy (Table 4).

**(Fig. 1: group A):**
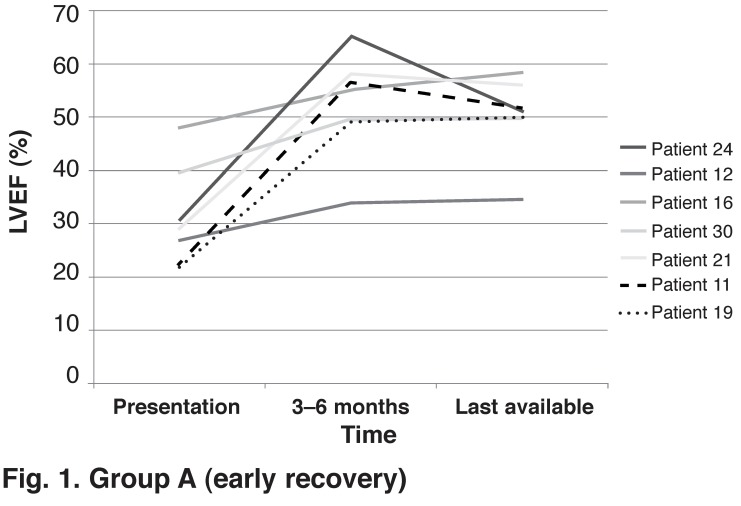
Group A (early recovery)

**(Fig. 2: group B):**
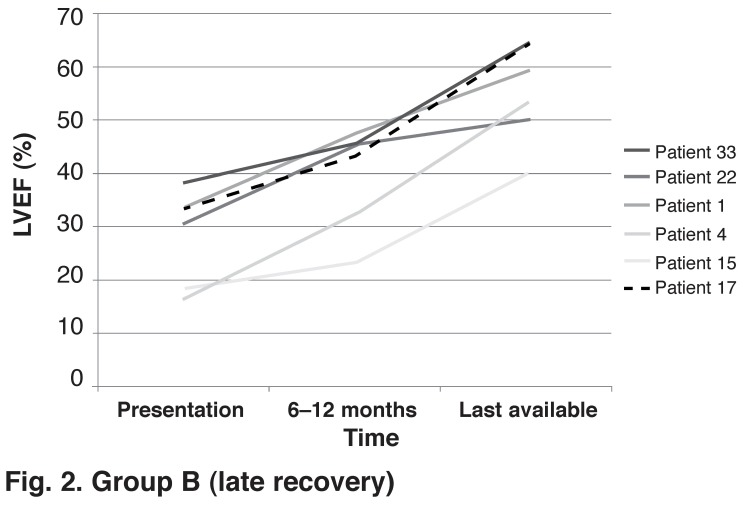
Group B (late recovery)

**Table 4:** Pure TIC Patients With Lenient Heart Rate Control And Late Improvement Of Left Ventricular Function

*Patient number*	*Average resting heart rate on treatment (beats/min)*
Patient 33	111
Patient 1	92
Patient 15	100
Patient 4	96

Seventeen patients with pure TIC who had an improvement in LVEF > 50% after control of the tachycardia were compared to 17 control patients with normal echocardiograms, matched for age and LVEF (Table 5). Patients with pure TIC had a trend of increased residual LVIDd dimensions compared to the control group, indicating a persistence of adverse LV remodelling late after control of the causative tachycardia (*p* = 0.06). There were no significant differences between the LVIDs and LA dimensions between the two groups.

**Table 5:** Post-Treatment Echocardiographic Parameters Of Patients With Pure TIC With Complete LV Improvement, And Normal Controls Matched For Age And LVEF

	*Pure TIC group (n = 17)*	*Normal controls (n = 17)*	p*-value^#^*
LVEF (%)	58.9 ± 8.9	60.4 ± 9.2	0.5
LVIDd (cm)	5.2 ± 0.6	4.8 ± 0.5	0.06
LVIDs (cm)	3.5 ± 0.5	3.2 ± 0.6	0.18
LA size (cm)	4.2 ± 0.7	3.9 ± 0.5	0.19

^#^Student’s *t*-test.

## Discussion

Our relatively large study of TIC has several important findings. This is the first study to compare patients with pure and impure TIC. Patients with impure TIC had shorter durations of dyspnoea and more clinical signs of heart failure at presentation. Our study supports the observation that patients with underlying structural heart disease may develop LV dysfunction more quickly and present earlier with symptoms and signs of heart failure.^04^

At initial presentation, TIC may be indistinguishable from DCMO with secondary tachycardia – the chicken–egg dilemma.^05^ Pure TIC patients had a better effort tolerance (NYHA class), complained of more palpitations and had fewer clinical signs of heart failure at initial presentation compared to patients with DCMO. Pure TIC patients also had fewer ECG conduction system abnormalities (with shorter QRS durations, and were less likely to have Q waves, LVH and repolarisation abnormalities) compared to patients with DCMO.

A previous study showed that certain electrocardiographic features may be useful for differentiating between DCMO and ischaemic CMO.^07^ DCMO was characterised by higher RV^06^ voltages and higher RV^06^/R^max^ (leads I, II or III) ratios. The authors stated that RV^06^ voltage is determined by the distance from the LV to the RV^06^ chest lead and is a marker of LV dilatation. Limb voltages in leads I, II, III are not affected by the distance of the LV to the chest wall and are markers of voltage-producing cardiac mass. In patients with DCMO, replacement fibrosis or infiltrates reduce R-wave voltages in these limb leads.

When we compared TIC patients with DCMO patients, there were no significant differences in RV^06^ voltages and RV^06^/R^max^ ratios between the two groups. By inference, RV^06^ voltages and RV^06^/R^max^ ratios may be useful parameters to distinguish DCMO or TIC from ischaemic CMO but not useful to distinguish between DCMO and TIC.

A previous echocardiographic study by Jeong *et al*. showed that TIC patients tended to have smaller LV mass indices, volume dimensions and LV dimensions compared to patients with DCMO.^08^ They suggested that a LVIDd ≤ 6.1 cm at presentation predicted TIC with a sensitivity of 100% and a specificity of 71%. Our study confirmed that pure TIC patients had significantly smaller LVIDd, LVIDs and LA dimensions when compared to DCMO patients at presentation. However, in contrast to their study, we could not confirm a LVIDd dimension that predicted TIC with a high degree of sensitivity and specificity.

In our study, we noted in four patients with AF (*n* = 2) and AT (*n* = 2) that although pharmacological rate control was suboptimal (according to the AFFIRM trial targets), LV function improved in all patients at a slower rate over a year. In contrast, we noticed that in patients who had AVNA and PPM implanted for failed pharmacological rate control, the LV function generally had maximal improvement by three to six months. These findings suggest that although strict rate control is required for optimal LV recovery, lenient rate control may still result in improvement.

In the RACE II trial,^09^ a randomised trial of optimal heart rate control versus lenient heart rate control in patients with permanent AF, researchers found no difference between the two strategies. However, TIC patients were under-represented in this trial. Currently, it seems that TIC patients may benefit from stricter heart rate control.

A previous study showed that although the LVEF improved significantly with control of the tachycardia, LV dimensions and volumes remained significantly elevated when compared to control patients late after control of the tachycardia.^10^ When we compared TIC patients who had complete improvement, with age and LVEF-matched control subjects with normal echocardiograms, TIC patients showed a trend of greater LVIDd dimensions, indicating persistence of adverse LV remodelling at follow up. LA dimensions also remained significantly elevated at follow up.

TIC patients with dilated ventricles at presentation were more likely to have residual LV dilatation at follow up. This observation stresses the importance of serial echocardiographic measurements at follow up. The clinical importance is that beta-blockers and renin–angiotensin system inhibitors should probably be prescribed to all TIC patients with residual LV dysfunction or dilated left ventricles at follow up, although data to support this recommendation are limited.

Nerheim *et al*. reported five patients with recurrent tachycardia, which caused a rapid decline in LV function and development of heart failure in all patients.^11^ These patients had an abrupt fall in LVEF within six months and reversed again within a similar period. Since that initial observation, another study reported two patients with recurrence of the tachycardia and TIC.^12^ Notably, these two patients had a very short period of symptoms from the tachyarrhythmia (12 days, one day) to presentation. Our study is the third report with recurrent tachycardias occurring in three patients.

Patient 41 developed an impure TIC secondary to an orthodromic AVRT with a previous inferior myocardial infarction. The presenting LVEF was 21%. He was started on metoprolol, enalapril and diuretics. At a three-week follow-up visit, his symptoms had markedly improved, with a repeat echocardiogram showing a LVEF of 51%, confirming a diagnosis of TIC. Unfortunately, he defaulted on medical therapy and re-presented with recurrence of the AVRT four months later. An echocardiogram confirmed that his LV function had deteriorated to a LVEF of 35%. His accessory pathway was successfully ablated. At two years’ follow up, he reported no recurrences of palpitations or AVRT. His last follow-up echocardiogram showed a LVEF of 48%. This case highlights the observation that recurrence of tachycardia may lead to a rapid decline in LV function.

Patient 4 developed a pure TIC secondary to two different ATs, with a presenting LVEF of 16%. Only one of the ATs could be ablated. Nevertheless, the LVEF improved to 61% one year after ablation. The patient was continued on anti-arrhythmia therapy. Eighteen months later, the first AT recurred, with deterioration in LVEF to 50% and mean heart rates on Holter of 119 beats/min. Pharmacological rhythm control was preferred. At the last follow up, the LVEF had improved to 53%.

Patient 23 developed a pure TIC secondary to rapid AFL with a presenting LVEF of 40%. While awaiting ablation of the AFL, the LVEF improved to 50% with pharmacological rate control. AFL ablation was unsuccessful. His AFL persisted and pharmacological AFL rate control (mean heart rates 97 beats/min) was noted to be suboptimal. This led to a slow progressive decline in LV function over three years (LVEF 32%). Successful AFL ablation led to improvement in LV function to a LVEF of 53% five months after ablation.

The long-term prognosis of TIC has not been established. Nerheim *et al*. documented three TIC patients who had recovered LV function and had sudden cardiac death.^11^ A study by Fujino *et al*.^13^ showed that cardiac death and recurrent hospitalisations were significantly less in the TIC group than the DCMO group.

In our study, 33 pure TIC patients were followed up for a mean period of 6.8 (range 1–19) years. Twenty patients were confirmed alive at the time of writing this article. There were three confirmed deaths. The nature of one death was due to a cholangiocarcinoma. The causes of death in the other two patients could not be determined from clinical records. Ten out of 33 patients could not be contacted. Of the 10 patients, four were discharged in a stable condition from the cardiac clinic. Three patients were discharged in a stable condition to other hospitals around South Africa. Three patients did not return for routine follow-up visits. We cannot exclude sudden cardiac deaths in patients who were not confirmed to be alive. In contrast, 14 out of 25 patients were alive in the DCMO control group. As a large number of TIC patients were lost to follow up, we could not make a meaningful comparison with DCMO patients regarding long-term prognosis and outcome.

## Limitations

This was a retrospective study with a small number of TIC patients. The patients represent a select group of non-consecutive patients referred to a regional referral hospital. It is likely that a sizeable number of patients with TIC were not included in this study. The true prevalence of TIC in patients with tachycardia can only be answered by a prospective study or CMO registry.

Although care was taken to examine all ECGs prior to presentation to calculate average heart rate, this may not be a true reflection of average heart rate during a 24-hour period. An accurate duration of the tachycardia before presentation was difficult to determine. Patients cannot always recall the exact duration of palpitations and patients could have a tachycardia for a variable length of time before an ECG is performed.

Not all patients with pure TIC had coronary angiography to exclude coronary artery disease, although no coronary intervention was performed between presentation and when LV function had recovered to account for improvement in LV function. Not all patients at presentation had echocardiography performed in sinus rhythm. The calculation of LV systolic function in the presence of a shortened diastolic filling time may result in an underestimate of LVEF.

In this study we were unable to control for the effects of differences in heart rate or rhythm in the assessment of LV function at initial presentation and after control of the tachycardia. Unfortunately, heart rate and rhythm at the time of the initial LV assessment at echocardiography were not routinely recorded on the echocardiography or radionucleotide imaging reports at our institution. However, echocardiographic reports at slower heart rates were preferentially recorded for LV functional assessment for better accuracy of LV function. When more than one echocardiogram was performed in short succession, an average of the LVEF and LV dimensions were calculated from serial echocardiograms to improve accuracy. In some cases, echocardiography was not performed at our institution. Echocardiography and interpretation of ECGs were often the interpretation of one cardiologist.

The small control group of 25 DCMO patients was limited by the number of DCMO patients available in the CMO registry. Treatment of tachycardia varied between cardiologists and was not administered uniformly. The long-term prognosis of TIC could not be accurately determined because a large number of patients were lost to follow up as they were not contactable. There was no standard follow-up time. Not all patients had repeat echocardiograms and echocardiograms were performed at variable time periods by different echocardiographers.

## Conclusions

This is one of the largest retrospective studies of TIC and the first large series from Africa. This study is the first to compare features of pure and impure TIC. Impure TIC patients have shorter duration and more severe symptoms at presentation. This study reports the first description of ECG findings of TIC. Patients with pure TIC have shorter QRS durations, fewer Q waves, and less LVH and repolarisation abnormalities at presentation compared to DCMO patients.

This is the second study to report that TIC patients have smaller LVIDd dimensions at presentation and have persistence of adverse LV remodelling as characterised by persistent enlargement of LVIDd dimensions at late follow up. Patients who have larger LVIDd dimensions at presentation tended to have larger LVIDd dimensions at late follow up, identifying a subgroup of patients where careful follow up is required.

This study confirms that maximal improvement of LV function generally occurs by three to six months. However, we noted slower improvement in LV function over 12 months in patients who had severe LV dysfunction and in patients with lenient heart rate control. We managed to identify 45 patients with this condition at a large regional referral centre in Cape Town over a 15-year period, suggesting that this condition is frequently under-diagnosed and missed.
